# Low–High–Low Rotationally Pulse-Actuated Serial Dissolvable Film Valves Applied to Solid Phase Extraction and LAMP Isothermal Amplification for Plant Pathogen Detection on a Lab-on-a-Disc

**DOI:** 10.1021/acsomega.3c05117

**Published:** 2024-01-09

**Authors:** Lourdes AN Julius, Muhammad Mubashar Saeed, Tim Kuijpers, Sergei Sandu, Grace Henihan, Tanja Dreo, Cor D Schoen, Rohit Mishra, Nicholas J Dunne, Eadaoin Carthy, Jens Ducrée, David J Kinahan

**Affiliations:** † Fraunhofer Project Centre at Dublin City University, Dublin City University, Glasnevin D09 V209, Dublin, Ireland; ‡ School of Physical Sciences, Dublin City University, Dublin D09 V209, Ireland; § National Centre for Sensor Research (NCSR), Dublin City University, Dublin D09 V209, Ireland; ∥ Biodesign Europe, Dublin City University, Dublin D09 V209, Ireland; ⊥ School of Mechanical and Manufacturing Engineering, Dublin City University, Glasnevin D09 V209, Dublin, Ireland; # SFI Centre for Research Training in Machine Learning (ML-Laboratories), Dublin City University, Dublin D09 V209, Ireland; ○ National Institute of Biology, 1000 Ljubljana, Slovenia; □ Wageningen University and Research, 6708 PB Wageningen, The Netherlands

## Abstract

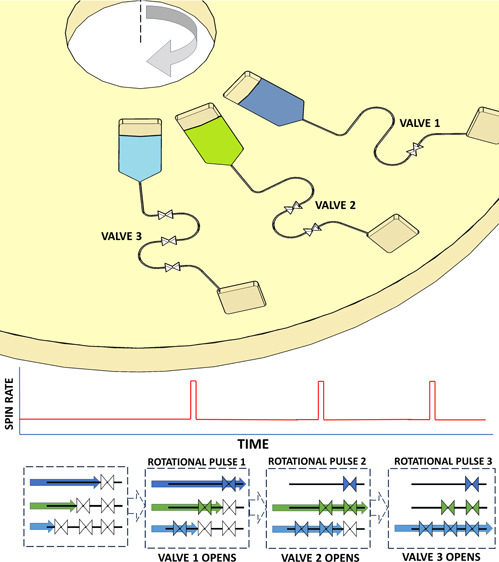

The ability of the centrifugal Lab-on-a-Disc (LoaD) platform to closely mimic the “on bench” liquid handling steps (laboratory unit operations (LUOs)) such as metering, mixing, and aliquoting supports on-disc automation of bioassay without the need for extensive biological optimization. Thus, well-established bioassays, normally conducted manually using pipettes or using liquid handling robots, can be relatively easily automated in self-contained microfluidic chips suitable for use in point-of-care or point-of-use settings. The LoaD’s ease of automation is largely dependent on valves that can control liquid movement on the rotating disc. The optimum valving strategy for a true low-cost and portable device is rotationally actuated valves, which are actuated by changes in the disc spin-speed. However, due to tolerances in disc manufacturing and variations in reagent properties, most of these valving technologies have inherent variation in their actuation spin-speed. Most valves are actuated through stepped increases in disc spin-speed until the motor reaches its maximum speed (rarely more than 6000 rpm). These manufacturing tolerances combined with this “analogue” mechanism of valve actuation limits the number of LUOs that can be placed on-disc. In this work, we present a novel valving mechanism called low–high–low serial dissolvable film (DF) valves. In these valves, a DF membrane is placed in a dead-end pneumatic chamber. Below an actuation spin-speed, the trapped air prevents liquid wetting and dissolving the membrane. Above this spin-speed, the liquid will enter and wet the DF and open the valve. However, as DFs take ∼40 s to dissolve, the membrane can be wetted, and the disc spin-speed reduced before the film opens. Thus, by placing valves in a series, we can govern on which “digital pulse” in spin-speeding a reagent is released; a reservoir with one serial valve will open on the first pulse, a reservoir with two serial valves on the second, and so on. This “digital” flow control mechanism allows the automation of complex assays with high reliability. In this work, we first describe the operation of the valves, outline the theoretical basis for their operation, and support this analysis with an experiment. Next, we demonstrate how these valves can be used to automate the solid-phase extraction of DNA on on-disc LAMP amplification for applications in plant pathogen detection. The disc was successfully used to extract and detect, from a sample lysed off-disc, DNA indicating the presence of thermally inactivated *Clavibacter michiganensis ssp. michiganensis (Cmm)*, a bacterial pathogen on tomato leaf samples.

## Introduction

Emerging challenges associated with climate change, loss of biodiversity, and overuse of antimicrobials has resulted in an emerging need for accurate detection of pathogens in humans, animals, and plants and the point-of-need/point-of-care.^1−7^ Portable diagnostic testing can result in the faster diagnosis of time-critical diseases such as sepsis and meningitis^8,9^ than centralized hospital laboratories. It can support correct interventions for neglected tropical diseases where testing infrastructure may otherwise not be available^10^ and can support management of emerging epidemics. In the area of food security, managing plant pathogens is an increasing concern. Pesticide resistance^11^ and the exposure of plants to pathogens that they may not have evolved resistance (due to either pathogen transmission through increased global connectivity or through crops planted new regions) is an increasing concern. It is clear there is a need for early detection of plant pathogens to support the best early interventions to control disease outbreaks.^12^

Lab-on-a-Chip offers the potential to address this need for detection of plant pathogens.^7,13−15^ A range of detection strategies have been applied to this challenge,^16−22^ including nucleic acid methods.^23−29^ The ability of the centrifugal Lab-on-a-Disc (LoaD)^30−33^ platform to mimic laboratory unit operations (LUOs),^32^ such as metering, mixing,^34^ and aliquoting, allows direct transfer, with minimal biological optimization, of established bioassays, normally conducted by hand or on liquid handling robots. A key benefit of this technology is the ability to accept samples by direct pipetting rather than needing complex loading and priming steps.^35,36^ These features make the LoaD platform particularly appealing for a wide range of point-of-care and point-of-use applications.^30−33,37,38^. Nucleic acid (NA) methods have been implemented on the LoaD by a number of research groups.^23,26−29,35,39−55^ LoaD has also been used for the detection of foodborne pathogens using NA methods.^26−29^

A key enabling technology on LoaD platforms is valving to control the LUOs in the correct sequence. A range of technologies have been applied to this, including “instrument supported” valves where the valve actuation is controlled by instrumentation provided in addition to a spindle motor for spinning the disc.^56^ These range from heat sources to ablate or melt open valve^57−60^ to integration of air pumps.^61^ While capable and reliable, these valve technologies are often expensive and complex. More common is to use changes in the disc spin-speed to open valves on the LoaD. This generally keeps the LoaD instrumentation for flow-control to a cheap, light, and low-cost spindle motor. However, valves that are defined by open channels in a microfluidic disc, such as capillary^62,63^ or siphon valves,^64^ are often unreliable and are usually limited to 3–4 LUOs. To circumvent this, a range of other valving strategies have been used, including integration of dissolvable films.^65^

The use of DFs on the LoaD typically involves integrating water-dissolvable films into the disc by recessing them into dead-end pneumatic chambers.^65^ By increasing the disc spin-speed, the reagent can be forced into the valve to wet the DF, thereby opening the valve. By tuning the size and geometry of the pneumatic chamber, we can tailor the spin-speed at which the valve opens. Modeling this using Boyle’s Law is facile,^65,66^ and through careful design, automation of 8 LUOs has been demonstrated using these valves.^66^ Both rotationally actuated “burst” DF valves^65^ and “event-triggered”^34^ dissolvable film valves have been applied to a wide range of applications^35,49,67−77^ with good success. However, a limitation of DF valves remains that the actuation of these valves is dependent on either tuning the valve opening spin-speed, which necessitates limiting the number of LUOs on the disc, or determining the dissolve time of the DF (usually ∼40 s), limiting the ability to perform sometimes assay critical incubations on the LoaD.

Recently, we introduced a new type of DF valve architecture called pulse-actuated DF valves^78^ which we applied to plant pathogen detection. With this technology, the order in which the valves are actuated is determined by the architecture of the disc through an integrated network of pneumatic channels. The valves are actuated by a low–high–low (LHL) pulse in disc spin-speed, which is freely programmable by the spindle motor. Thus, the timing of the LUOs can be varied and optimized rather than being dependent on DF dissolve time. While an advancement in the state-of-the-art, these pulse-actuated valves have several disadvantages. They require a significantly large dead volume, which results in lost reagents and, more critically, lost sample. Additionally, the complex pneumatic channels must link the outlet of one reservoir to the valve restraining liquid in the next reservoir. This can lead to complex channel geometries that can be difficult to design and manufacture.

In this work, we present a novel DF valve architecture that enables LHL pulse actuation of valves without requiring a network of connecting pneumatic channels. In these valves, a DF membrane is placed in a dead-end pneumatic chamber. Below an actuation spin-speed, the trapped air prevents the liquid wetting and dissolving the membrane. Above this spin-speed, the liquid will enter and wet the DF and open the valve. However, as DFs take ∼40 s to dissolve, the membrane can be wetted, and the disc spin-speed is reduced before the film opens. Thus, by placing valves in series, we can govern on which “digital pulse” in spin-speed a reagent is released; a reservoir with one serial valve will open on the first pulse, a reservoir with two serial valves on the second, and so on. This “digital” flow control mechanism allows automation of complex assays with high reliability.

We will first describe the operation of the valves and outline the theoretical basis for their operation and support this analysis with experiment. Critically, we demonstrate both theoretically and experimentally that for reliable operation there must be an air vent between each sequential valve. Next, we demonstrate how these valves can be used to automate solid-phase extraction of DNA and on-disc loop-mediated isothermal amplification (LAMP) for applications in plant pathogen detection. LAMP is particularly suitable detection of plant pathogens at for point-of-use as it requires a single temperature to amplify and is highly sensitive and specific.^14,79−81^ This is demonstrated through the detection, from a sample lysed off-disc, of DNA indicating the presence of thermally inactivated *Clavibacter michiganensis ssp. michiganensis (Cmm)* bacterial pathogen on tomato leaf samples

## Valve Operation

### Theory of Valve Operation

The theoretical operation of DF burst valves have previously been described,^65,66^ but will be reiterated here both for convenience of the reader and to ensure a consistent nomenclature. The nomenclature will be adapted from that used by Mishra et al.^66^

This theoretical analysis is important, as it demonstrates that venting of trapped air between sequential valves, or indeed not venting air between sequential valves, will have an impact on subsequent valve opening frequencies.

The centrifugally induced hydrostatic pressure, Δ*P*_c_, is defined as
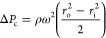
1where ρ is the density of the liquid, ω is the angular velocity of the disc in radians, *r*_o_ is the radially outer position of the liquid element relative to the center of rotation, and *r*_i_ the radially inward location of the liquid element relative to the center of rotation. Therefore, Δ*P*_c_ is the pressure difference measured between *r*_o_ and *r*_i_.

Note that an alternative nomenclature is sometimes used where Δ*P*_c_ is defined as Δ*P*_c_ = ρω^2^Δ*rr̅*. In this case, Δ*r* is the radial length of the liquid element (i.e., Δ*r* = *r*_o_ – *r*_i_) and *r̅* is the central radial location of the liquid element (i.e., ).

**Figure 1:**
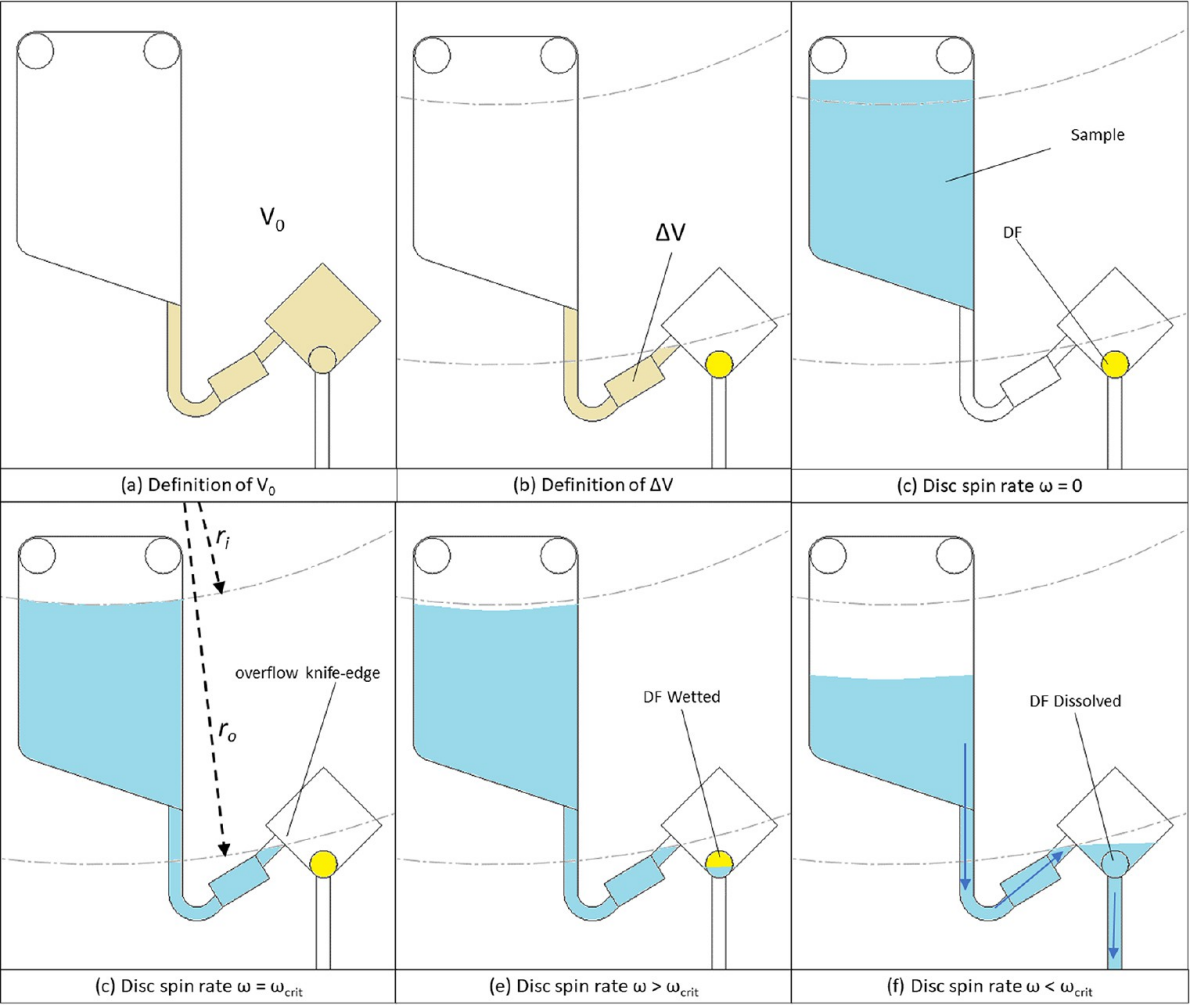
Schematic showing valve operation.

The DF burst valves operate based on the principle that air trapped in a dead-end pocket, sealed with a DF membrane, will prevent liquid from entering and then wetting/dissolving a DF membrane. However, above a critical spin rate, ω_crit_, the centrifugally inducted hydrodynamic pressure will compress the gas within the pocket, letting the liquid impinge and wet the DF (Figure 1). The valve architecture used in this study, proposed by Dimov et al.^82^ and optimized by Mishra et al.,^66^ uses a u-shaped microchannel to place the heavy liquid below the lighter air in the valve structure. This architecture minimizes the effects of surface tension and makes the valves more reliable and predictable at higher disc spin-rates.^66^ In this architecture, shown in Figure 1, the pneumatic chamber can be divided into two parts. The entire valve has a volume of *V*_0_, while the section of the valve that must be filled before the DF is wetted is Δ*V*.

If the disc is stationary when loaded and the liquid sample blocks the inlet of the valve (Figure 1c), the gas trapped in the valve is at atmospheric pressure *P*_0_. With the disc in rotation, centrifugally induced hydrodynamic pressure Δ*P*_crit_ is required to compress the trapped air to volume *V*_crit_, where *V*_crit_ = *V*_0_ – Δ*V*. Thus, from Boyle’s Law:
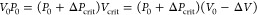
2Substituting eq 1 into eq 2,
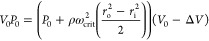
3Of these parameters, *P*_0_ and ρ are environmental and material properties, while *r*_o_, *V*_0_, and Δ*V* are design parameters which are a function of the disc architecture. Similarly, while *r*_i_ can be varied in some cases by changing the volume of sample loaded on a disc, it can be assumed to be a constant based on the disc operating under design parameters. The disc angular velocity, ω, is the operational parameter that is changed to actuate the valve. Therefore, writing eq 3 in this term gives
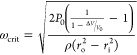
4Equation 4 applies to a single burst valve or indeed sequential burst valves if they are vented to the atmosphere between each valve structure. However, when valves are placed in series and not vented, the critical burst frequency will be highly dependent on the air pressure at which upstream valves are actuated.

Taking the case of two sequential valves where the disc is manufactured at atmospheric pressure *P*_0_. The first valve is open to atmosphere during loading and then becomes a sealed pneumatic chamber in the presence of the sample. The second valve starts with the pressure *P*_0_ and volume *V*_0_.

In an alternative variation of eq 2, the gas pressure inside the first valve in series on opening (bursting) can be defined as
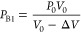
5On dissolving the DF, this pressure will be equalized through the second burst valve. The first valve has pressure *P*_B__1_ and volume *V*_0_ – Δ*V*. The second valve has a pressure *P*_0_ and a volume *V*_0_. After pressure is equalized, the liquid in the valve splits the first valve from the second valve. Thus, for the purposes of calculating its burst pressure, the starting pressure in the second valve in sequence, *P*_0,2_ can be defined as
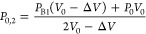
6and, thus, the burst pressure of V2 is
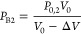
7Therefore, the burst pressure of the next unvented valve (i.e., V3) will be impacted on the burst pressure calculated for the preceding valve (V2), and this will be the case for all valves in a series.

**Figure 2:**
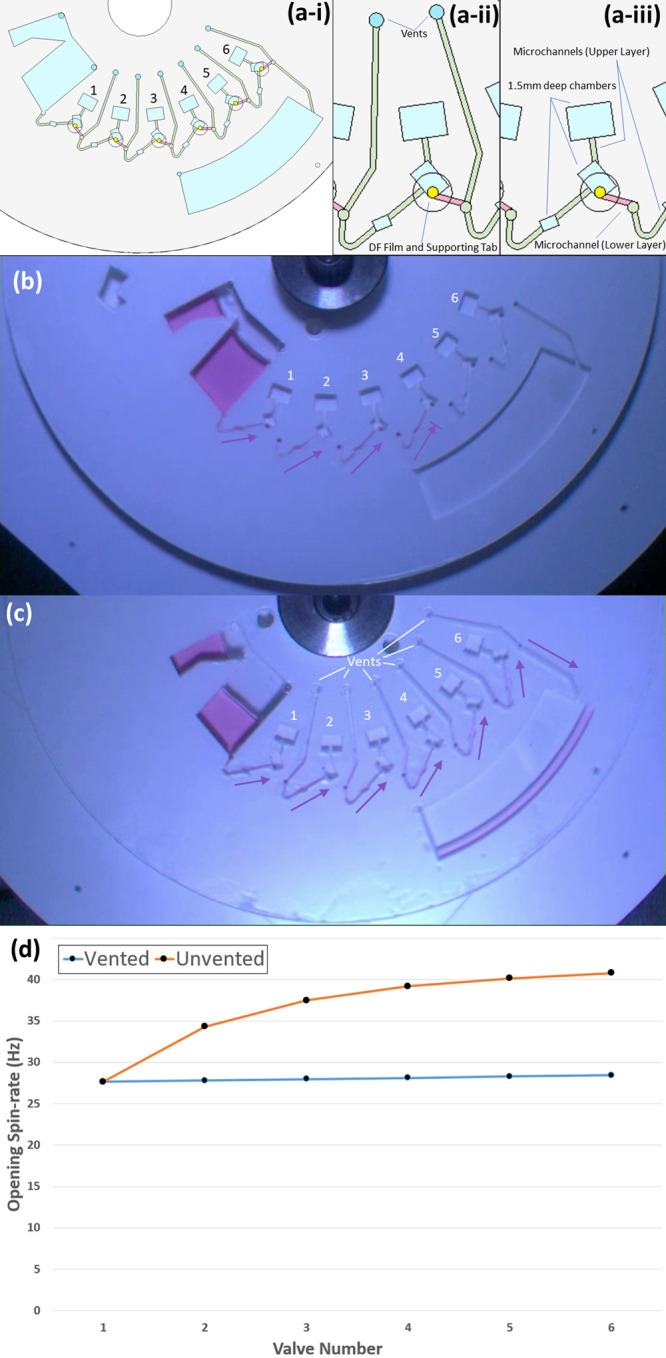
Impact of venting on valve opening spin-rates (a-i) shows schematic of the disc with 6 sequential vented valves, (a-ii) a detailed view of the one of the vented valves, and (a-iii) a detailed view of one of the unvented valves. Note the color coding represents features of different depths on different layers in the disc (b) shows an image of the disc tested with 6 sequential vented valves. (c) An almost identical architecture where there are air vents between each valve. (d) Calculated burst frequencies using eqs 6 and 7 to calculate the burst frequencies based on the architectures shown in (b) and (c). See also SI, Movie 1 and Movie 2.

To demonstrate the cumulative effect on burst frequency of not venting valves, a disc was created with two identical structures. These structures were composed of an inner chamber, six sequential DF burst valves, and a waste reservoir. Six vents were then integrated into the second structure, as shown in Figure 2a–c. Based on the disc architecture, each theoretical burst pressure (and therefore burst spin frequency) was calculated using eqs 6 and 7. The displacement of liquid into the valves, which has an effect on *r*_i_, was considered, but other potential sources of error, such as dead volumes, were not considered. Figure 2d shows the theoretical burst frequencies calculated for vented and unvented architectures. The values vary from V1 at 27.7 Hz to V6 (unvented) at 28.4 Hz and V6 (vented) at 40.8 Hz.

In Figure 2, the disc is loaded with dyed water (which is metered to a defined volume by the architecture of the loading chamber), and the disc is spun at a continuous rate of 40 Hz. In the case of the unvented structure (Figure 2a), V1–V3 open, but V4 does not open, while for the vented structure (Figure 2b) all valves are open. This broadly supports the calculated frequencies presented in Figure 2c, where it is estimated that V1–4 should open. Note that the test shown in Figure 2 was repeated in triplicate, and in each case, V1–3 opened, but V4 did not open. A video of this test is shown in SI, Movie 1 and Movie 2.

### Principle of Valve Operation

**Figure 3:**
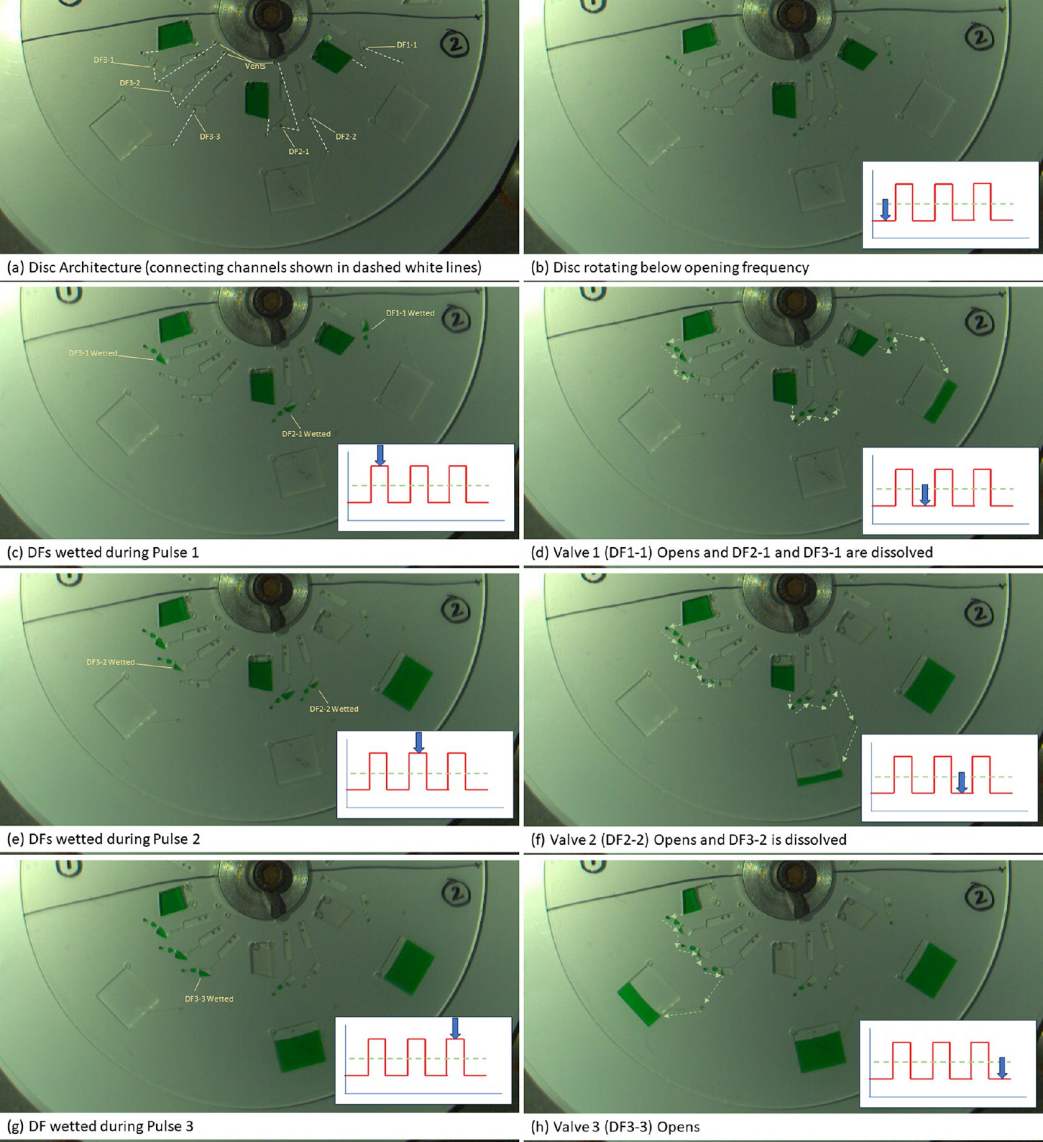
Demonstration of 3 valves each designed to actuate on a different pulse: (a) shows the disc architecture showing a valve which will open after 1 pulse (DF1), after two pulses (DF2), and after 3 pulses (DF3); (b)–(h) show the sequence of valve actuation. Critically, as the DFs take ∼30 s to dissolve, assuming each pulse is less than 30 s in duration, only one DF in each valve will be wetted and dissolved on each pulse. See also SI, Movie 3.

To demonstrate the principle of operation of the Low-High-Low serial pulse valves, Figure 3 shows a disk architecture with three reservoirs. Each reservoir is valved by three valves, the first which opens after one pulse (i.e., one valve), the second opens after two pulses (i.e., two serial valves), and the third after three pulses (i.e., three serial valves). These valves were designed to open at 40 Hz spin-rate. The disc was rotated at 30 Hz during normal operation and during low-high-low pulses was accelerated at 40 Hz s^–1^ to 50 Hz. After 10–12 s the disc is decelerated (again at 40 Hz s^–1^ to 30 Hz). During this time, the DF film is wetted but does not dissolve/disintegrate until ∼30 s have passed (at which time the disc is spinning at 30 Hz below the design burst frequency of the next valve in series).

For the demonstration disc shown in Figure 3, during the first pulse (Figure 3c), Valve 1 (DF1-1) is wetted, as is the first DF in Valves 2 (DF2-1) and 3 (DF3-1). On reducing the spin-speed is reduced below the actuation frequency (Figure 3d), these DFs remain wetted and eventually dissolve. This opens Valve 1 and allows the liquid to move through Valve 2 and Valve 3 (Figure 3d). On the next pulse, DF2-2 is wetted (opening Valve 2), and DF3-2 is wetted and dissolved (allowing liquid to move through Valve 3). On the third pulse, Valve 3 is opened.

## Materials and Methods

### Plant Pathogen Assay

#### Assay Primers

In this study, we present an integrated disc that screens a sample against six LAMP target primers. These targets are an internal control, *cytochrome oxidase gene (COX)*, with primers adapted from Tomlinson et al.,^83^ bacteria target *Clavibacter michiganensis ssp. michiganensis (Cmm)* from Yasuhara-Bell et al.,^84^ RNA virus *Pepino mosaic virus (PepMV)* from Hasiow-Jaroszewska et al.,^85^ the viroid target *Potato Spindle Tuber Viroid (PSTVd)* from Lenarcic et al.,^86^ the fungus *Botrytis cinerea (BOTRY)* from Tomlinson et al.,^87^ and fungal blight *Phytophthora infestans (P. INF)* was adapted from Hansen et al.^88^ The primers were purchased from IDT (Leuven, Belgium) and validated against G-blocks (also acquired from IDT). The primers and G-blocks have previously been made available.^78^

The benchtop and on-disc protocols have also been described previously.^78^ Briefly, reactions of 25 μL volumes were created from primers (4 μL) and LAMP reagent ISO-100 (Optigene, U.K.; 15 μL). The total volume was reached through the addition of “samples” of either 6 μL of buffer containing DNA (for positive controls) or 6 μL of nuclease-free water (for negative controls). In all reactions, the samples contained bovine serum albumin (BSA, Sigma-Aldrich, U.K.) at a concentration such that the final concentration of BSA in the reaction was 1%. BSA was required for on-disc testing to block the surfaces of the microfluidic device and prevent the adsorption of DNA/key reagents. Isothermal amplification was performed on a commercial qPCR instrument (Qiagen Rotorgene, Manchester, U.K.; 60 min, 65 °C, SYBR Green acquisition every 10 s), followed by melt curve analysis.

The disc architecture was designed such that a total volume of 30 μL was used in each reaction chamber. Ten μL of this volume was preloaded into the amplification chambers and was made up of primers (9 μL) and mineral oil (1 μL). The additional 20 μL was delivered from the metering structure. This was composed of a 4:1 ratio of the LAMP reagent and sample. The LAMP reagent had BSA added, such that the final assembled volume of the aqueous sample contained 1% BSA.

#### Plant Sample Preparation

The tomato leaves are prepared using the DNEasy Plant Mini Kit (Qiagen) using a protocol adapted from Mishra et al.^78^ Briefly, 70 mg of plant material is cooled in liquid nitrogen and ground to powder using a mortar and pestle. At this point, if the sample is spiked with thermally inactivated CMM bacteria (IPO-3208), a 53 μL volume (diluted to the appropriate concentration) is added.

Next, a lysis buffer and RNase are added, and the sample is incubated for 10 min at 65 °C. A second buffer is then added. Next, the sample is incubated on ice for 30 min. The sample is then further processed through spin-columns, provided as part of the kit, using supplied buffers. Because the DNEasy kit includes the addition of an RNase, amplification from PepMV and PSTVd (which are RNA viruses and viroid, respectively) was not expected. In this study, only LAMP amplification chemistry (rather than RT-LAMP) was used. Once the DNEasy protocol was completed, for on-bench controls, sample purification (Qiagen QiaQuick) was performed as per the instructions of the manufacturer.

In the case of on-disk testing, once the DNEasy protocol was completed, the sample was centrifuged. Then, 33 μL of supernatant was mixed with 167 μL of PB Buffer (from the Qiagen QiaQuick kit), and a total of 200 μL was loaded on-disc.

#### Centrifugal Test Stand

The discs were tested on a centrifugal test-stand as previously described by Mishra et al.^78^ Briefly, the test-stand uses a spindle motor (Festo EMME-AS-55-M-LS-TS, Esslingen, Germany) which is synchronized with a scientific camera (Basler Ace 2040-90uc, Basler, Germany) and stroboscopic light source (Drelloscop 3244, Drello, Germany) using an external trigger signal. This permits videos to be created for flow visualization purposes where the disc is rotating but appears stationary. The basic test-stand is further modified with a heating system and a fluorescence detection system as described previously.^78^ The instrument is controlled using a custom program (written in LabVIEW, National Instruments, Texas, U.S.A.), which has full control of the instrument, including the heating system (temperature, clamping, unclamping), the disc (speed, acceleration, positioning), and fluorescence detector (laser on/off, acquire measurement). This permits the tests to be conducted entirely autonomously once the discs are loaded; however, for these experiments, only the amplification steps take place autonomously. Note that further details of the experimental test-stand are provided in the SI.

#### Disc Fabrication

The discs fabricated for this study were assembled from laser cut PMMA (Vink König, Gilchin, Germany) and knife-plotter cut pressure sensitive adhesive (PSA; ARCare 7840, Adhesives Research, Limerick, Ireland) using the methods described previously.^35^ Briefly, the discs are 120 mm (demonstration discs, Figures 2 and 3) or 160 mm (plant pathogen disc) and are designed in SolidWorks (Dassault Systems, Paris, France). The discs are assembled manually on a custom assembly jig and rolled in a high-pressure laminator during assembly (HL-100, Cheminstrument, U.S.A.). The PMMA layers are cut on a laser cutter (Epilog Xing (EpilogLaser, Colorado, U.S.A.)), and the PSA layers are cut with a knife plotter (Graphtec CE6000) Graphtec Corporation, Tokyo, Japan). If the discs are intended for biological testing, the PMMA layers are prepared using washing/sonication, as described previously.^35^ The DF tabs were prepared as described previously^35^ and were made of KC35 film (Aciello, Japan), which has a dissolve time of 30–40 s.^35^ However, PE buffer/EtoH (Qiagen QiaQuick kit) does not dissolve KC35 at the desired concentrations. Therefore, a custom dissolvable film (Adhesives Research, Limerick, Ireland) was used for these specific tabs.^78^ This is referred to as AR film and dissolves in ∼20 s in even high concentrations (up to 95%) of EtOH (though it does not dissolve in 100% EtoH). The disc layers are defined in Table 1.

**Table 1:** Layers Used for Disc Assembly

name	material	function
layer 1	PMMA (0.5 mm)	Top layer of disc and contains loading vents.
layer 2	PSA (0.086 mm)	Contains reservoirs, microchannels and pneumatic venting channels.
layer 3	PMMA (1.5 mm)	Reservoirs for liquids.
layer 4	PSA (0.086 mm)	Provides additional sealing around DF tabs. Layers 4 and 5 also contain additional microchannels and pneumatic venting channels.
layer 5	PSA (0.086 mm)	Support layer for alignment/placement of DF tabs. Layers 4 and 5 also contain additional microchannels and pneumatic venting channels.
layer 6	PMMA (1.5 mm)	Reservoirs for liquids (particularly when the reservoir extends from layer 2 to layer 6 to create 3 mm depth reservoirs). Support for DF tabs during assembly.
layer 7	PSA (0.086 mm)	Contains microchannels and pneumatic venting channels. Microchannels on this layer permit “crossing” of channels to allow greater flexibility in design.
layer 8	PMMA (0.5 mm)	Lower layer (base) of the disc.

Before testing, the solid-phase (acid washed glass beads, (Sigma-Aldrich)) is added to the disc using the method described previously.^49,78^ The end of a standard 1000 μL plastic pipet tip is trimmed to make the opening larger and make it into a funnel. It is aligned with an opening in the disc and filled with beads; the pipet tip is tapped gently, and the beads are gravity fed into the disc. With the chamber filled, the pipet is removed, and the opening is sealed with transparent PSA (Adhesives Research, Limerick, Ireland).

#### Disc Operation

As described above, prior to testing, the lysate (purified by the DNEasy Plant Mini Kit (Qiagen)) was centrifuged, and 33 μL of supernatant was mixed with 167 μL of PB Buffer (from the Qiagen QiaQuick kit). For operation, the disc is loaded with 200 μL of PE buffer for washing the solid phase, 200 μL of EB Buffer for elution, and 80 μL of EB Buffer to act as an ancillary liquid. It was also loaded with 160 μL of LAMP reagent. As each chamber is loaded, it is sealed with transparent PSA. As described above, the ability to vent the serial burst tubes is critical for their performance. In contrast, it is critical that discs where DNA amplification occurs are sealed entirely from the atmosphere to ensure no laboratory contamination (with high concentrations of amplified DNA targets). This design criterium was met by ensuring that all chambers were linked by pneumatic venting channels so air pressure could equalize within the disc while the disc, in its entirety, was sealed from atmosphere.

Next, the six amplification chambers were loaded with 1 μL of mineral oil and 9 μL of target primers and again sealed from the atmosphere. As these chambers were either dead-end chambers or sealed with DF valves, the disc could be rotated to help with the loading of these reagents. However, the last two reagents were sealed with capillary valves; therefore, it was critical that they be loaded in parallel without rotating the disc during these steps. There were loading 10 μL of FC-40 (Sigma-Aldrich, Ireland) to enable immiscible liquid valve the routing structure^49^ and loading the 200 μL sample. Both chambers are sealed, and the test protocol is started.

**Figure 4:**
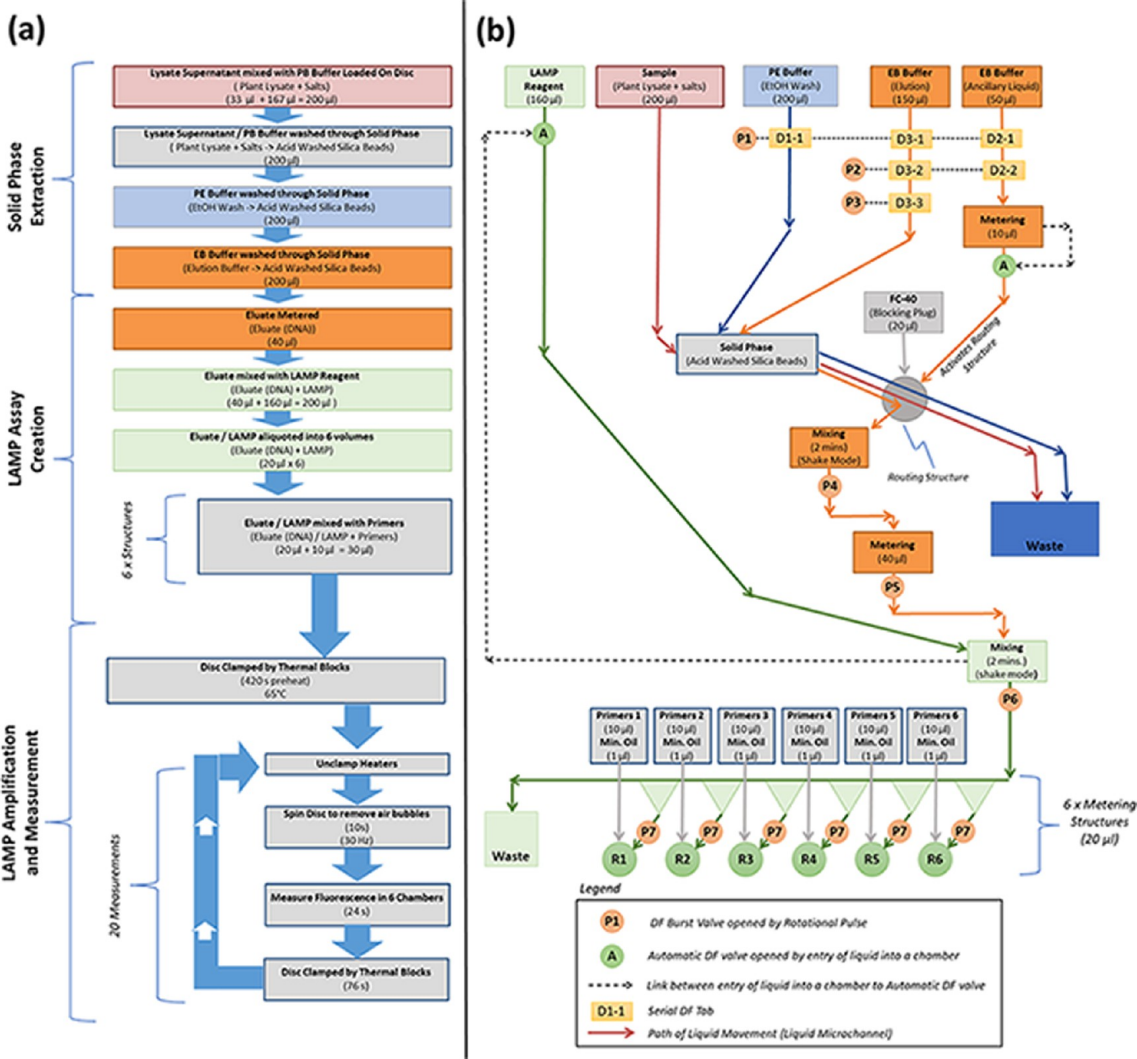
Schematics describing (a) the individual LUOs automated on the disc and (b) the architecture and relationship of low–high–low pulses to reagent release. Valves labeled “P#” are opened by the corresponding low–high–low digital pulse shown in the motor spin-rate profile illustrated in Figure 5. Valves labeled “A” are opened by the presence of liquid in a reservoir and the link between liquid entry and valve actuation is highlighted by a dashed line. The subcomponents of the serial valves are labeled “D#-#”. This refers first to the total number of valves in a series and then to the specific valve number in the series. For example, D3-1 refers to the first valve of three serial valves. When D3-3 is wetted and opened (in this case by P3), the reagent will be released.

**Figure 5:**
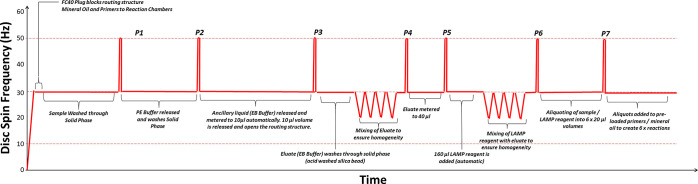
Spin protocol used to automate the on-disc assay.

**Figure 6:**
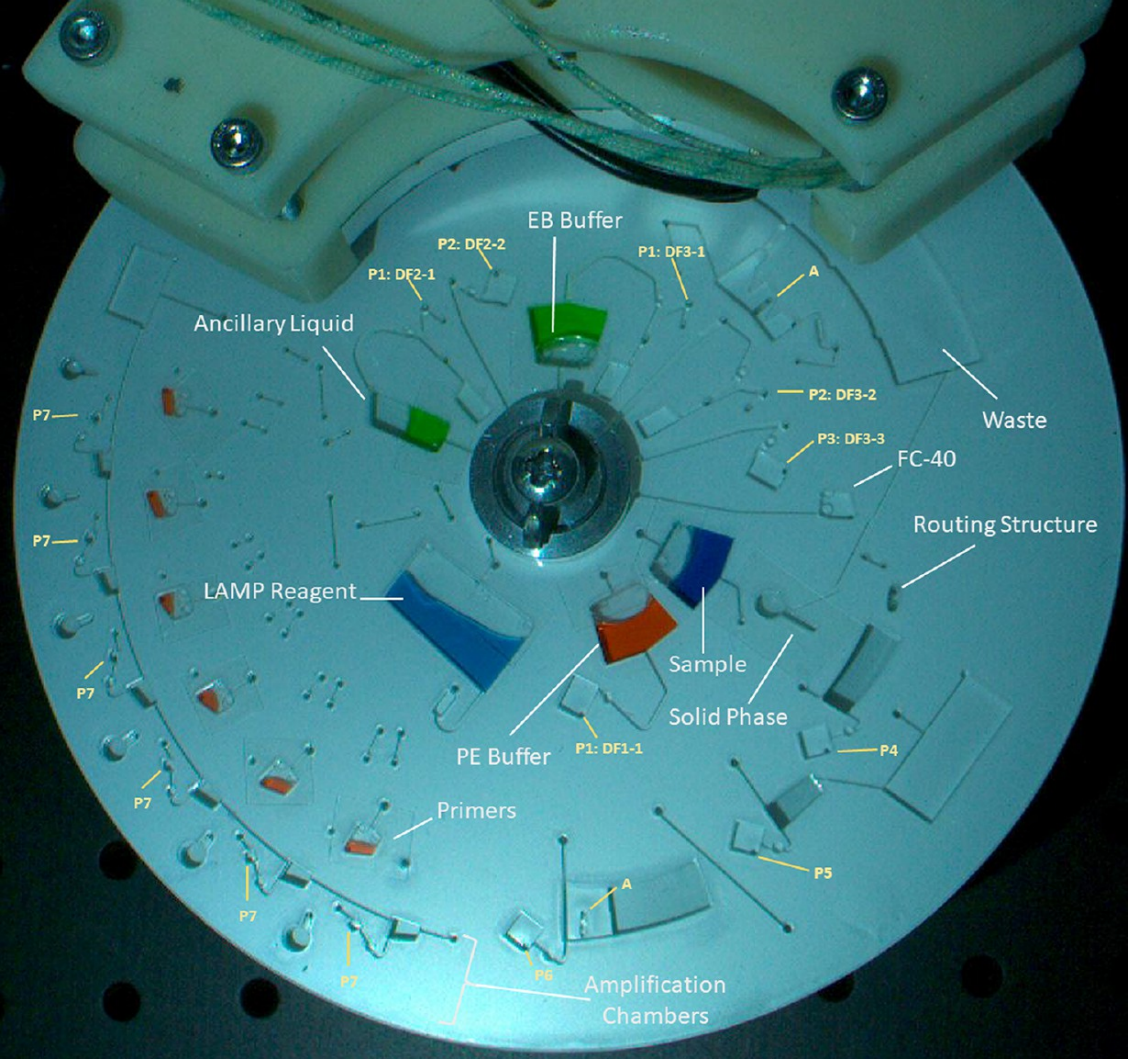
Image of the disc used for plant pathogen detection. The disc is loaded with colored water for visualization in this case and is designed to be fully sealed from the atmosphere. Reagents loaded on the disc are indicates. Serial pulse valves are indicated as DF1-1, etc., and DF burst valves by P4, etc. SI, Movies 4 and 5 show the full operation of this disc. SI, Movies 6 and 7 show the operation of the SPE with no heater blocks present.

The test protocol is defined here and illustrated in Figures 4 and 5, while the disc architecture is shown in Figure 6. Note that the disc is accelerated and decelerated at 40 Hz s^–1^ during experiments and stays at the higher spin rate for 10 s, unless otherwise stated. The tests were run manually, with the disc performance monitored using the stroboscopically coupled camera.

- The disc is rotated at 30 Hz. The FC-40 is pumped into the routing structure to block a DF film with an immiscible plug of liquid.^49^ The primers and mineral oil are pumped into the amplification chambers. The sample flows through a capillary valve through the column of acid washed glass beads and into the waste chamber.
- Pulse 1 (increasing spin-speed to 50 Hz for 10 s and then returning the spin-speed to 30 Hz) opens DF1-1 (AR film), which releases the PE buffer wash. The films DF2-1 and DF3-1 are also dissolved.
- Pulse 2 (increasing spin-speed to 50 Hz for 10 s and then returning the spin-speed to 30 Hz) opens DF2-2 and releases the ancillary liquid (EB buffer). This liquid is metered to a small volume (10 μL) using an automatic metering structure, which is enabled by an event-triggered DF valve (labeled A).^35^ This smaller volume then flows over the routing structure and opens it. DF3-2 is also dissolved.
- Pulse 3 (increasing spin-speed to 50 Hz for 10 s and then returning the spin-speed to 30 Hz) opens DF3-3 and releases the elution buffer EB buffer. This is washed over the solid phase and routed to a collection chamber. Next, the disc is accelerated and decelerated (20–30 Hz) to mix and homogenize the eluate.
- Pulse 4 (increasing spin-speed to 50 Hz for 10 s and then returning the spin-speed to 30 Hz) opens valve labeled P4 and allows the eluate to follow into a metering chamber. The eluate is metered to 40 μL. Note that this valve, and all subsequent valves, do not open on early pulses as these chambers are empty during these pulses.
- Pulse 5 (increasing spin-speed to 50 Hz for 10 s and then returning the spin-speed to 30 Hz) opens the valve labeled P5 and transfers the metered eluate into a mixing chamber. Here, an event-triggered DF, labeled A, is dissolved, which mixes 160 μL of LAMP reagent with the eluate. This mixing is enhanced by accelerating and decelerating (20–30 Hz) the disc.
- Pulse 6 (increasing spin-speed to 50 Hz for 10 s and then returning the spin-speed to 30 Hz) opens the valve labeled P6, which allows the LAMP/DNA to flow into the 6 × 20 μL metering structures.
- Pulse 7 (increasing spin-speed to 50 Hz for 10 s and then returning the spin-speed to 30 Hz) opens the valves labeled P7, which transfers 20 μL of LAMP/DNA into each of the amplification chambers.

#### On-Disc Amplification

For on-disc LAMP amplification, the first reaction chamber is manually aligned with the fluorescence detector, and the motor location is set to zero. Next, a preprogrammed script is activated, which controls the motor and heater unit. The lights are turned out, and the instrument runs autonomously. The script commands the instrument to clamp the disk for 720 s to heat the reaction chambers. After this, the following cycle occurs every 110 s:

1. Disc is unclamped and the motor powers/enabled.
2. Disc rotates at 30 Hz for 10 s to remove any air bubbles that may have appeared in the reaction chambers.
3. The disc aligns with each reaction chamber and takes a fluorescence measurement. This takes 4 s per reaction chamber (24 s total).
4. The reaction chambers are aligned with the thermal block, and the motor depowers.
5. The disc is clamped, and the chambers are heated.

During this sequence, the thermal block is unclamped for ∼35 s and heated for ∼75 s. Due to the insulating properties of plastic, the reaction temperature only drops by ∼1 °C during the ∼35 s measurement window.^78^ For the experiments presented in this paper, the script was set to make 20 measurements. Including the 420 s preheat, the entire amplification sequence took ∼45 min. The on-disc SPE and reaction creation took ∼15 min, resulting in lysate-to-answer in approximately 1 h.

## Results and Conclusions

**Figure 7:**
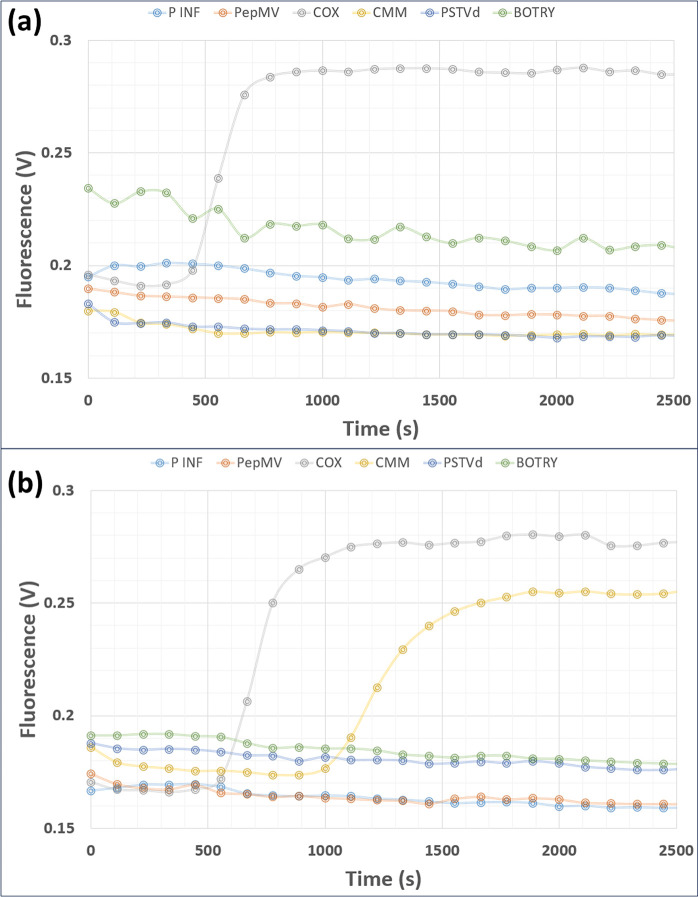
LAMP amplification curves acquried from the Lab-on-a-Disc using the custom laboratory instrument (a) shows amplification curves from a tomato leaf sample and (b) shows amplification curves from a tomato leaf sample, which was spiked by 10^7^ CFU/mL of thermally inactivated CMM bacteria. Note that the 420 s preheat is not included on the time axis.

Fluorescence amplification curves were successfully acquired from lysed plant pathogen samples and lysed plant pathogen samples, which were spiked with thermally inactivated CMM bacteria (Figure 7) and clearly demonstrate the ability of the system to identify the presence of CMM. Overall, this work demonstrates the capability of these novel serial dissolvable film burst valves to automate a complex assay using a low–high–low digital rotational spin profile. We demonstrate their operation and support this through theoretical analysis of their operation with a particular focus on demonstrating the importance of venting these valves to maintain a reliable and consistent opening frequency. These valves are shown to function reliably when embedded into discs manufactured using a low-cost but low-fidelity assembly technique. Thus, these valves will be even more reliable if used in discs manufactured using higher fidelity techniques such as milling or injection molding. Similar to the valves first introduced by Mishra et al.,^78^ these valves have an advantage in instrumentation in that they only require two spin-rates to operate (low and high), and so are compatible with low-cost spindle motors. The timing of valve actuation is dependent on the timing of the digital pulse in spindle speed, while the order of actuation depends on the disc architecture (i.e., how many DFs are placed in series in each valve).

These valves have been used to demonstrate a complex lysate-to-answer protocol incorporating DNA cleanup and the creation of 6-plex LAMP assays with on-disc amplification. While these tests took place on a laboratory test-stand not suitable for deployment in the field, as these valves can be actuated only by a spindle motor using only two spin-speeds, it is clear they are highly suitable for deployment at point-of-use/point-of-care using portable laboratory instruments. On bench testing using this protocol indicated positive amplification from plant material (COX) after ∼12 min and from 10^7^ CFU/mL of CMM after ∼16 min. By comparison, on-disc the amplification of plant control (COX) took ∼16 min and the 10^7^ CFU/mL of CMM took ∼24 min. This difference likely reflects a combination of the time taken for the disc to reach amplification temperature and a reduction in on-disc SPE efficiency (i.e., packed acid washed beads compared to the silica frit in commercial spin columns). It should also be noted that the concentration of bacteria used, 10^7^ CFU/mL is relatively high. It concentration chosen as the innovation focus of this work is demonstrating complex liquid handling protocols using novel valve technology. Further work will be required to validate the capability of this system to detect lower bacterial concentrations. Similarly, our system operates using samples that have been lysed on-bench using standard protocols that cannot be easily automated in a microfluidic device. However, some emerging lysis chemistries^89,90^ or thermal lysis^91^ (particularly if thermal lysis is combined with serial dilution to reduce the impact of amplification inhibitors while leveraging the sensitivity/specificity of LAMP) are more amenable to use in microfluidic devices. Using these lysis strategies on a centrifugal disc, in conjunction with these novel valves, can potentially enable a true sample-to-answer capability.

The valves presented in this work function in a manner similar to those introduced by Mishra et al.^78^ and are demonstrated using a mostly identical assay. However, we identify that each valve type offers key advantages and disadvantages. The pulse-actuated (PA) valves presented by Mishra et al.^78^ can use a largely identical architecture for each valve. Their operation depends on a secondary DF, located in the path of a previous release of liquid, to dissolve before the valve can open. This hand-shaking mechanism can enhance their reliability but conversely means that a failure in a noncritical part of the disc (i.e., a waste chamber not filling to a desired volume due to a design error) might result in failure of an otherwise functional disc. In the case of the serial valves presented here, the valves are designed to actuate on a specific pulse and are not dependent on the proper operation of the upstream valves. Therefore, they can be conceptually considered individual valves (designed to open on a specific pulse), while the PA valves from Mishra et al.^78^ might be considered a single continuous architecture.

In another advantage, the serial valves presented here are conceptually simpler to understand and design than the PA valves. Critically, they do not require pneumatic channels connecting locations on the disc located radially inward and outward, thus simplifying disc design. These valves and these discs proved to be quite robust and reliable. Despite being a relatively complex architecture, which was assembled manually (albeit by an experienced researcher), once the design was finalised we observed failure in less than 20% of tested discs. The serial valves have a smaller dead volume and use less disc real estate when designed to operate on the first one, two, or three pulses. However, for each additional ’pulse’ on which the valves are designed to operate, the architecture uses more disc real estate and has an increasing dead-volume. Overall we recommend the use of these serial valves for simpler assays (i.e., requiring 3 or 4 LUOs) while recommend the PA valves of Mishra et al.^78^ for automation of more complex assays.

## Supporting Information Available

Data from benchtop controls and additional information regarding the performance of the centrifugal test-stand (PDF)
Movie 1: Annotated movie of experiment demonstrating benefits of venting serial DF valves (see Figure 2) (MP4)
Movie 2: Edited (split screen) annotated movie of experiment demonstrating benefits of venting serial DF valves (see Figure 2) (MP4)
Movie 3: Annotated movie showing actuation of sequential valves (see Figure 3) (MP4)
Movie 4: Annotated movie showing the operation of an integrated disc (see Figure 6) (MP4)
Movie 5: Movie (without annotation) showing the operation of an integrated disc (see Figure 6) (MP4)
Movie 6: Annotated movie showing the simplified disc used to demonstrate SPE (MP4)
Movie 7: Movie (without annotation) showing the simplified disc used to demonstrate SPE (MP4)
