# Supplementary material for: Low–High–Low Rotationally Pulse-Actuated Serial Dissolvable Film Valves Applied to Solid Phase Extraction and LAMP Isothermal Amplification for Plant Pathogen Detection on a Lab-on-a-Disc
Source: ACS Omega. 2024 Jan 9;9(3):3262–75. doi: 10.1021/acsomega.3c05117 (PMC10809376; doi:10.1021/acsomega.3c05117)
Supplement: Supplementary file 1 — ao3c05117_si_001.pdf [file ao3c05117_si_001.pdf]

Low-High-Low Rotationally Pulse-actuated Serial Dissolvable Film Valves applied to Solid Phase Extraction and LAMP Isothermal Amplification for Plant Pathogen detection on a Lab-on-a-Disc

Lourdes AN Julius[a,b,c,1], Muhammad Mubashar Saeed[d,e,f], Tim Kuijpers[d,e,2], Sergei Sandu[d,e], Grace Henihan[a,b,c], Tanja Dreo[g], Cor D Schoen[h], Rohit Mishra[a,b,c,3], Nicholas J Dunne[d,e], Eadaoin Carthy[c,d,e], Jens Ducrée[b,c,d] and David J Kinahan[c,d,e*]

[a] Fraunhofer Project Centre at Dublin City University, Dublin City University, Glasnevin, Dublin, Ireland
[b] School of Physical Sciences, Dublin City University, Dublin, Ireland
[c] National Centre for Sensor Research (NCSR), Dublin City University, Dublin, Ireland
[d] Biodesign Europe, Dublin City University, Dublin, Ireland
[e] School of Mechanical and Manufacturing Engineering, Dublin City University, Glasnevin, Dublin, Ireland
[f] SFI Centre for Research Training in Machine Learning (ML-Labs), Dublin City University, Dublin, Ireland
[g] National Institute of Biology, Ljubljana, Slovenia
[h] Wageningen University Research, Wageningen, The Netherlands

*Corresponding Author: David.kinahan@dcu.ie

[1] Karlsruhe Institute of Technology, Karlsruhe, Germany
[2] Eindhoven University of Technology Eindhoven, Noord-Brabant, NL 5600 MB
[3] Abbott Rapid Dx International, Ballybrit, Galway, Ireland

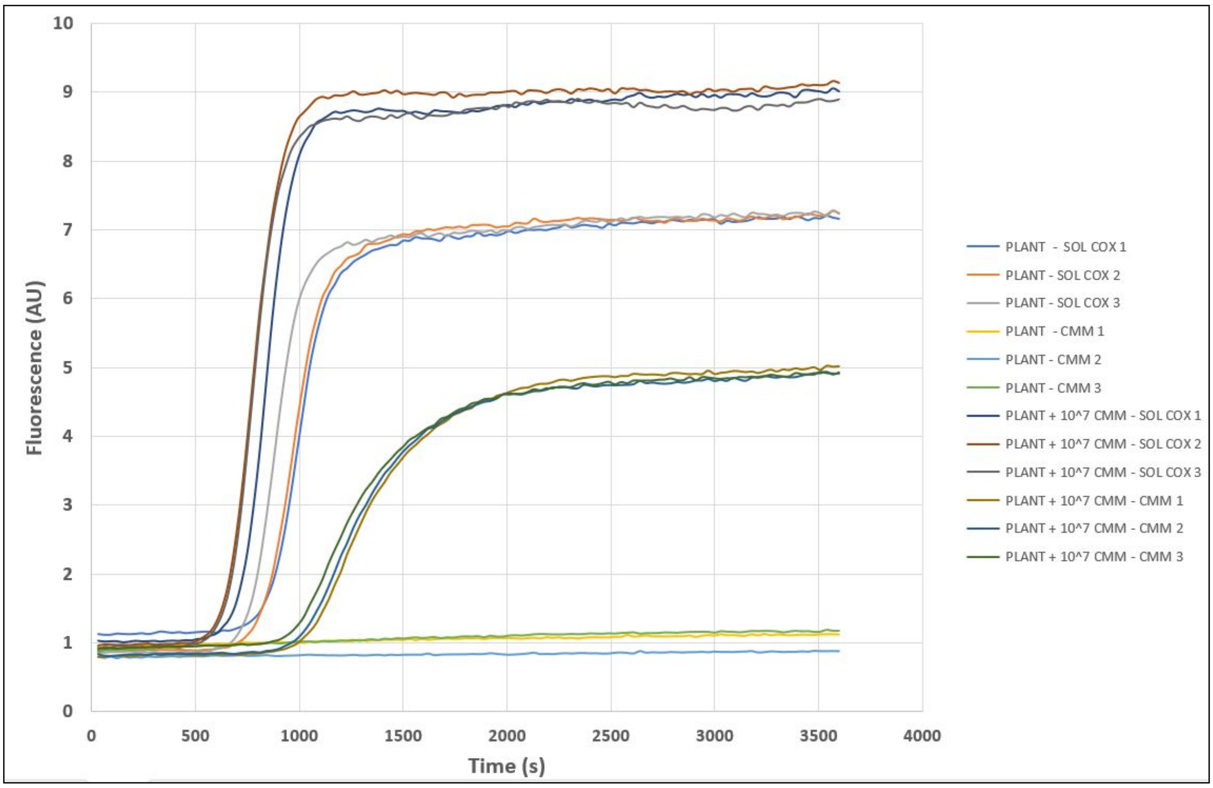

Figure S1: LAMP Amplification Curves from benchtop control experiments. Samples were prepared as per the paper but purified on-bench using Qiagen spin-columns. Then 20 µL LAMP samples were prepared and amplified in a commercial Qiagen Rotor-gene PCR machine with measurements every 30 s. Plant / SOL COX and Plant + 10^7CFU CMM / SOL COX amplified after ~ 12 minutes while Plant + 10^7CFU CMM / CMM amplified after ~ 16 minutes. As expected, Plant / CMM did not amplify. Note that earlier on-bench work for this paper was previously presented [1].

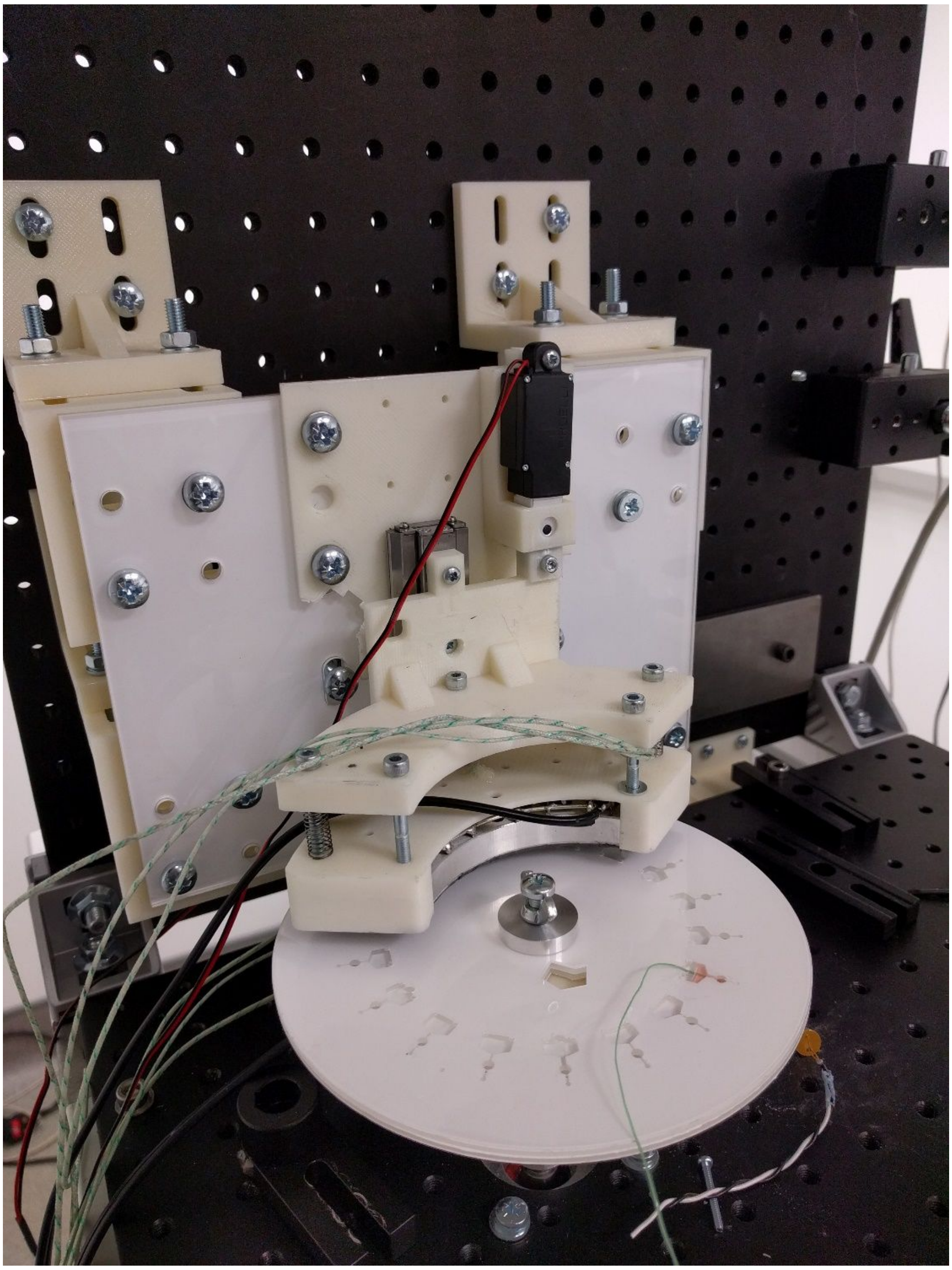

Figure S2: Image of the thermal blocks which clamp the disc top and bottom to heat it to temperature. Note a thermocouple embedded in a liquid filled reservoir can be seen in this image. Figures S3-S5 were measured using this thermocouple. Readers are referred to previous work which more fully describes the disc operation [1,2]. Figure adapted from Kinahan et al [1]

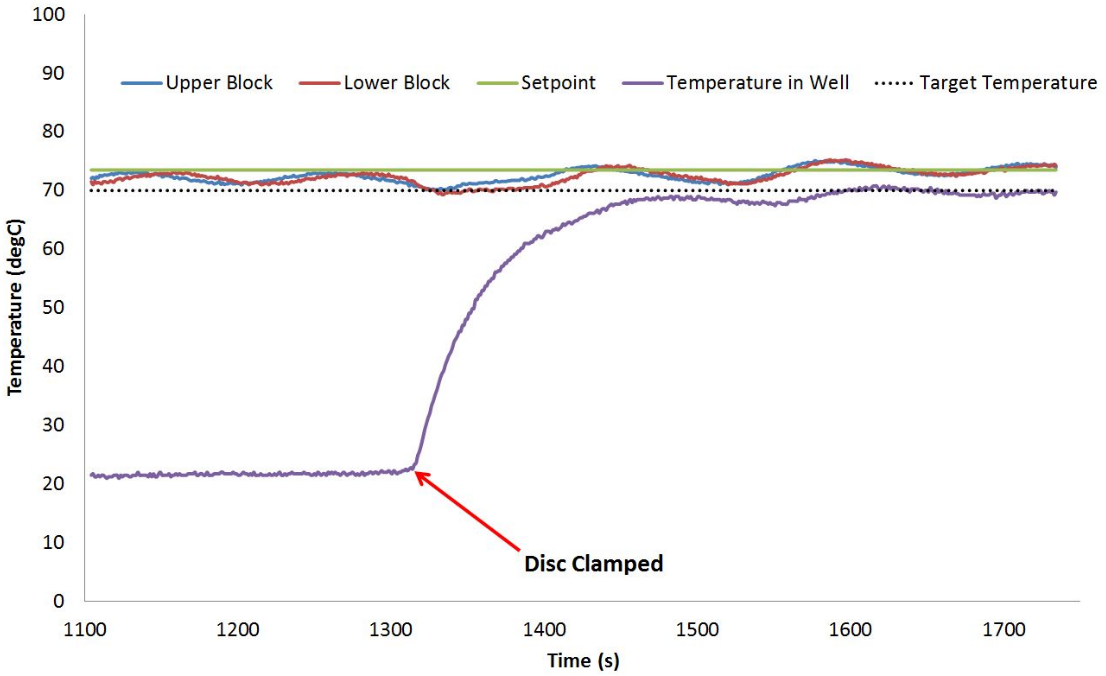

Figure S3: Temperature data indicating the response of temperature in a well to the discs clamping. It takes ~3.5 minutes for the samples to reach temperature. However, a 7-minute delay is used during experiments before the first fluorescence measurement. Note these measurements were made at 70 °C but LAMP amplification took place at 65 °C. Note in this case the thermal blocks were pre-heated prior to clamping. Figure adapted from Kinahan et al [1]

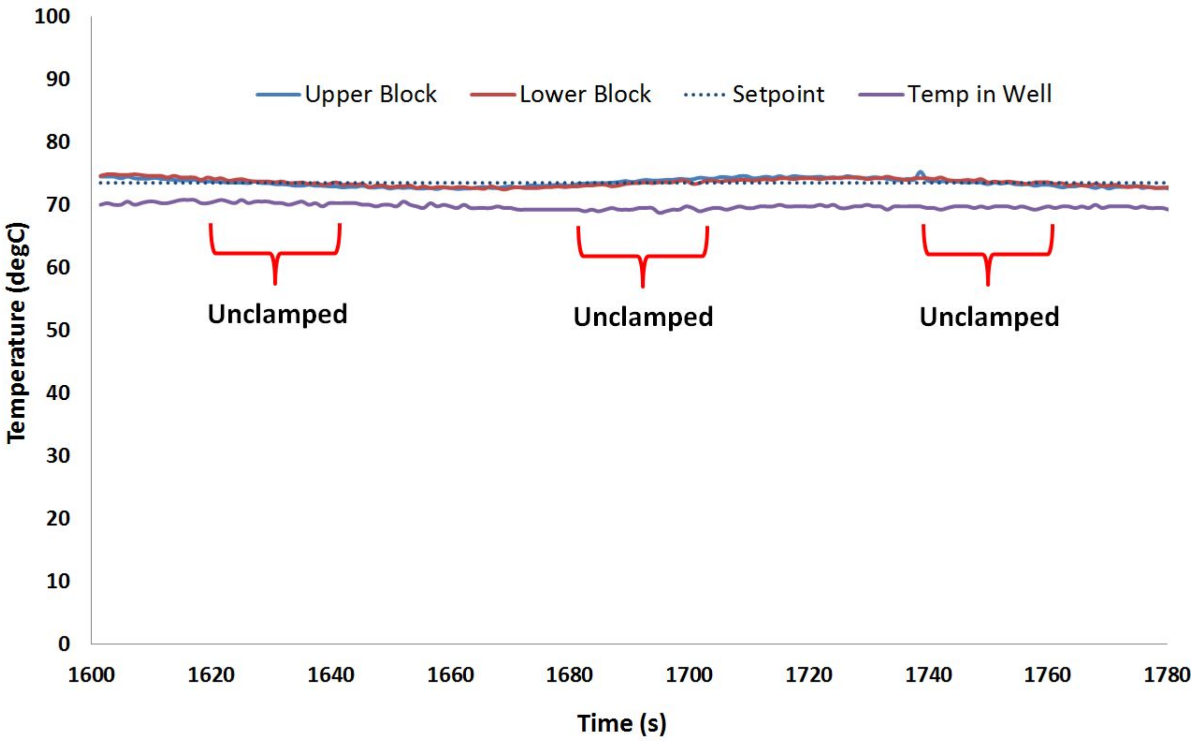

Figure S4: Temperature response inside a reservoir during unclamping of the disc for ~20 s showing no drop in temperature inside the reservoir. Similar testing for the 34 s required for fluorescent measurements similarly showed no measurable temperature drop. This is largely due to the thermal mass of the PMMA form which the disc is manufactured.

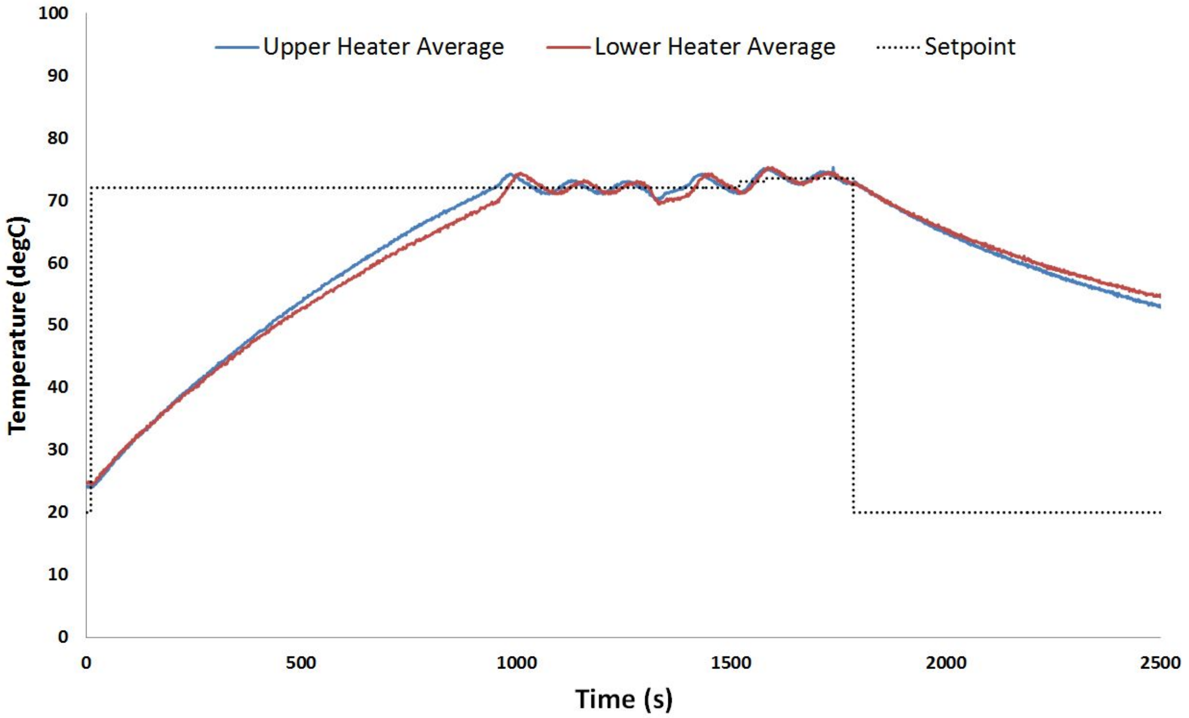

Figure S5: The time taken for the blocks to reach temperature. This takes ~17 minutes and in view of this delay the on-disc experiments took place with the discs pre-heated to the set-point (ensuring that the reagents in the disc reached temperature in ~3.5 minutes as shown in Figure S3. Figure adapted from Kinahan et al [1]

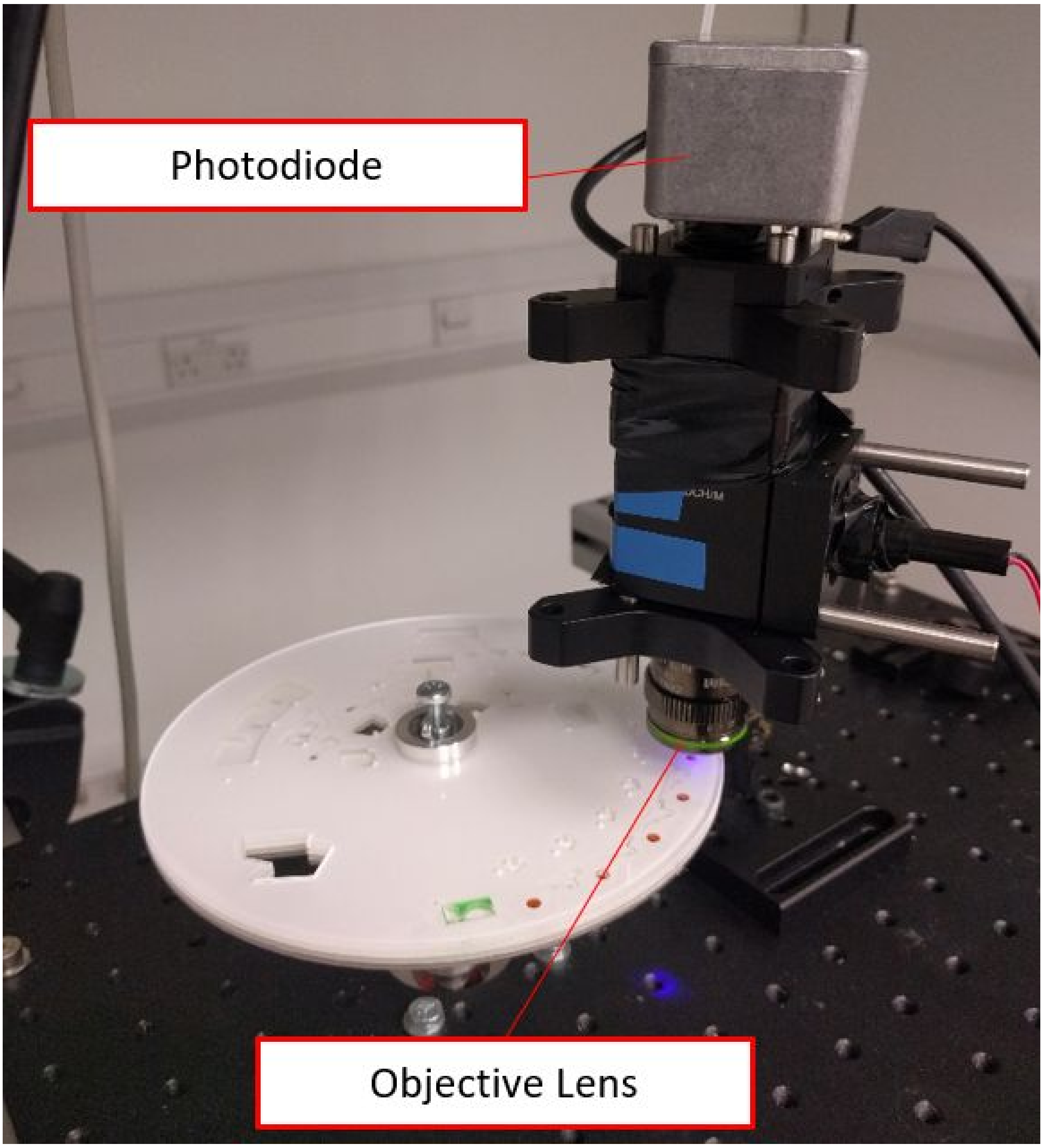

Figure S6: Image of the fluorescence detection system mounted above the disc. Figure adapted from Kinahan et al [1]

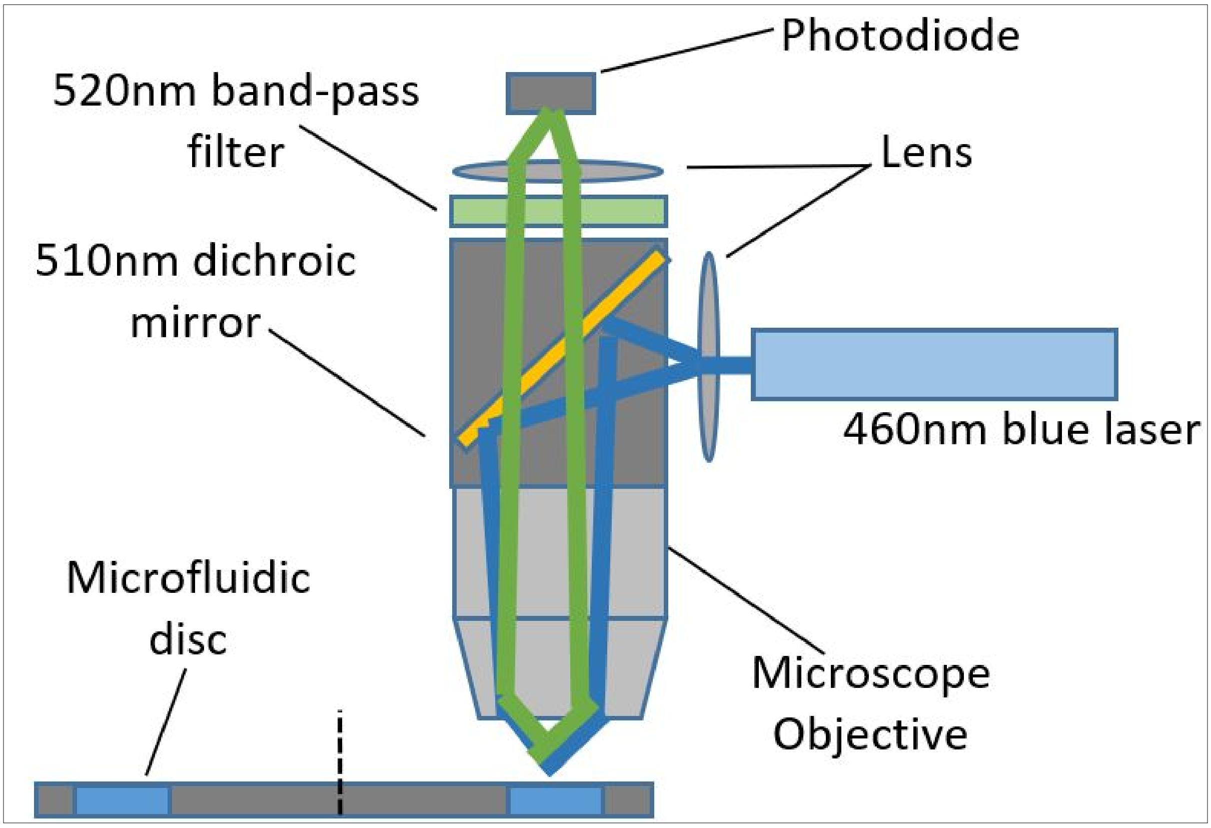

Figure S7: Schematic of the fluorescence detection configuration. Briefly, a low-cost 460nm is expanded and directed via a dichroic mirror and 10x microscope lens to the sample. It is defocussed when it illuminates the sample resulting in a largely diffuse and uniform excitation light field. Fluorescence emitted by the sample is focussed by the same 10x microscope and directed through the dichroic lens and then focussed onto an amplified detector photodiode. Figure adapted from Kinahan et al [1]

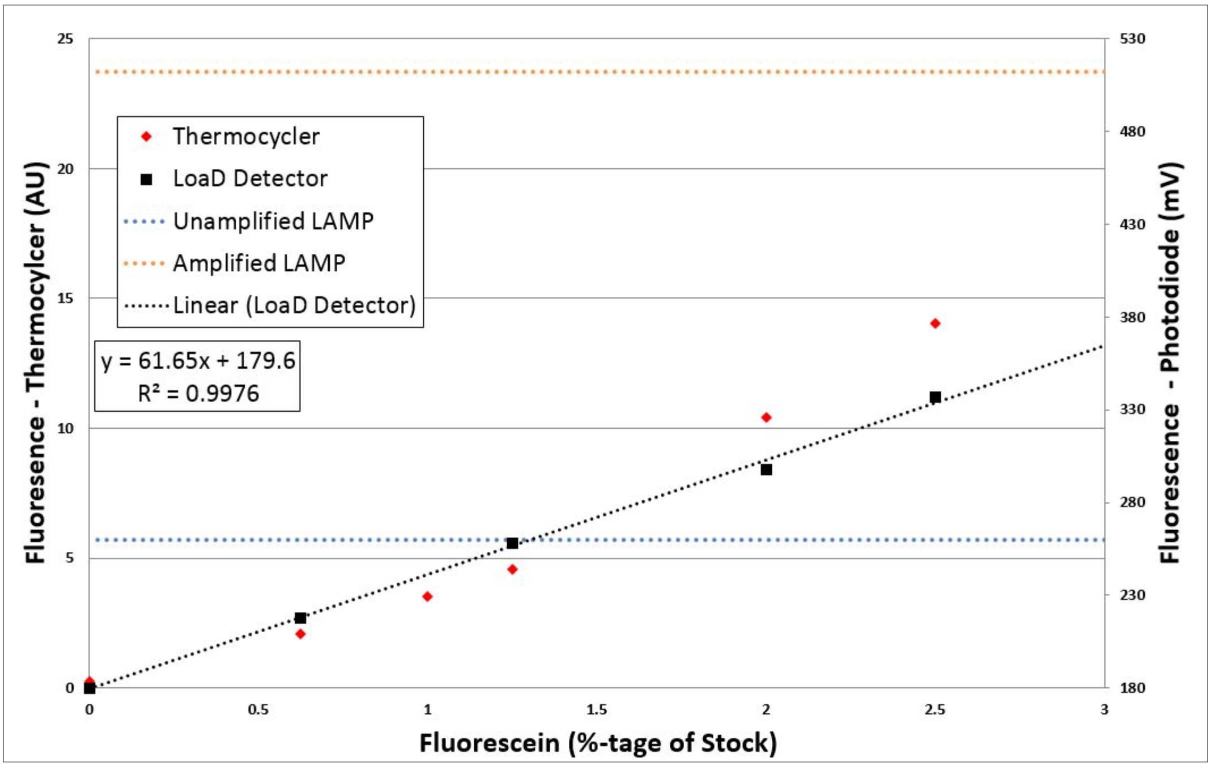

Figure S8: Calibration data for the fluorescence sensor. First, amplified, and unamplified LAMP reagent, and a serial dilution of fluorescein fluorescence dye were measured in a commercial PCR thermocycler (Qiagen Rotorgene)(left vertical axis). Fluorescent dye was tested in the same concentration using the fluorescence detector and the centrifugal disc (right vertical axis). The axes were scaled to align the fluorescein fluorescence dye measurements to a best fit. Results indicate that the photodiode demonstrates greater linearity and can detect and discriminate fluorescence signals lower than background (unamplified LAMP). Not that actual on-disc samples had signals from 160mV (NTC / unamplified) to 300mV (amplified) which likely indicates slight misalignment of the fluorescence detector compared to the results presented here. Figure adapted from Kinahan et al [1]

Additional information:

Experiments were run using custom control software (LabVIEW) which ran on human editable / readable script files to control the motors, heaters, and fluorescence detectors during amplification. Generally, the user monitored the disc during the assay steps (using a stereoscopically coupled camara) and manually entered the spin-protocol defined in Figure

References

(1) Kinahan, D. J.; Julius, L. A.; Schoen, C.; Dreo, T.; Ducrée, J. Automated DNA Purification and Multiplexed Lamp Assay Preparation on a Centrifugal Microfluidic “Lab-on-a-Disc” Platform; IEEE, 2018; pp 1134–1137.
(2) Mishra, R.; Julius, L. A.; Condon, J.; Pavelskopfa, P.; Early, P. L.; Dorrian, M.; Mrvova, K.; Henihan, G.; Mangwanya, F.; Dreo, T. Plant Pathogen Detection on a Lab-on-a-Disc Using Solid-Phase Extraction and Isothermal Nucleic Acid Amplification Enabled by Digital Pulse-Actuated Dissolvable Film Valves. *Analytica Chimica Acta* **2023**, *1258*, 341070.
